# BindsNET: A Machine Learning-Oriented Spiking Neural Networks Library in Python

**DOI:** 10.3389/fninf.2018.00089

**Published:** 2018-12-12

**Authors:** Hananel Hazan, Daniel J. Saunders, Hassaan Khan, Devdhar Patel, Darpan T. Sanghavi, Hava T. Siegelmann, Robert Kozma

**Affiliations:** Biologically Inspired Neural and Dynamical Systems Laboratory, College of Computer and Information Sciences, University of Massachusetts Amherst, Amherst, MA, United States

**Keywords:** GPU-computing, spiking Network, PyTorch, machine learning, python (programming language), reinforcement learning (RL)

## Abstract

The development of spiking neural network simulation software is a critical component enabling the modeling of neural systems and the development of biologically inspired algorithms. Existing software frameworks support a wide range of neural functionality, software abstraction levels, and hardware devices, yet are typically not suitable for rapid prototyping or application to problems in the domain of machine learning. In this paper, we describe a new Python package for the simulation of spiking neural networks, specifically geared toward machine learning and reinforcement learning. Our software, called BindsNET^1^, enables rapid building and simulation of spiking networks and features user-friendly, concise syntax. BindsNET is built on the PyTorch deep neural networks library, facilitating the implementation of spiking neural networks on fast CPU and GPU computational platforms. Moreover, the BindsNET framework can be adjusted to utilize other existing computing and hardware backends; e.g., TensorFlow and SpiNNaker. We provide an interface with the OpenAI gym library, allowing for training and evaluation of spiking networks on reinforcement learning environments. We argue that this package facilitates the use of spiking networks for large-scale machine learning problems and show some simple examples by using BindsNET in practice.

## 1. Introduction

The recent success of deep learning models in computer vision, natural language processing, and other domains (LeCun et al., 2015) have led to a proliferation of machine learning software packages (Jia et al., 2014; Abadi et al., 2015; Chen et al., 2015; Tokui et al., 2015; Al-Rfou et al., 2016; Paszke et al., 2017). GPU acceleration of deep learning primitives has been a major proponent of this success (Chetlur et al., 2014), as their massively parallel operation enables rapid processing of layers of independent nodes. Since the biological plausibility of deep neural networks is often disputed (Stork, 1989), interest in integrating the algorithms of deep learning with long-studied ideas in neuroscience has been mounting (Marblestone et al., 2016), both as a means to increase machine learning performance and to better model learning and decision-making in biological brains (Wang et al., 2018).

Spiking neural networks (SNNs) (Maass, 1996, 1997; Kistler and Gerstner, 2002) are sometimes referred to as the “third generation” of neural networks because of their potential to supersede deep learning methods in the fields of computational neuroscience (Wall and Glackin, 2013) and biologically plausible machine learning (ML) (Bengio et al., 2015). SNNs are also thought to be more practical for data-processing tasks in which the data has a temporal component since the neurons which comprise SNNs naturally integrate their inputs over time. Moreover, their binary (spiking or no spiking) operation lends itself well to fast and energy efficient simulation on hardware devices.

Although spiking neural networks are not widely used as machine learning systems, recent work shows that they have the potential to be. SNNs are often trained with unsupervised learning rules to learn a useful representation of a dataset, which may then be used as features for supervised learning methods (Diehl and Cook, 2015; Kheradpisheh et al., 2016; Ferr et al., 2018; Hazan et al., 2018; Saunders et al., 2018). Trained deep neural networks may be converted to SNNs (Rueckauer et al., 2017; Rueckauer and Liu, 2018) and implemented in hardware while maintaining good image recognition performance (Diehl et al., 2015), demonstrating that SNNs can in principle compete with deep learning methods. In similar lines of work (Hunsberger and Eliasmith, 2015; Lee et al., 2016; O'Connor and Welling, 2016; Huh and Sejnowski, 2017; Mostafa, 2018; Wu et al., 2018), the popular back-propagation algorithm (or variants thereof) has been applied to differentiable versions of SNNs to achieve competitive performance on standard image classification datasets, providing additional evidence in support of the potential of spiking networks for ML problem solving. Finally, ideas from reinforcement learning can be used to efficiently train spiking neural networks for object classification or other tasks (Florian, 2007; Mozafari et al., 2018).

The membrane potential (or voltage) of a spiking neuron is often described by ordinary differential equations. The membrane potential of the neuron is increased or decreased by *presynaptic* inputs, depending on their sign and strength. In the case of the leaky integrate-and-fire (LIF) model (Kistler and Gerstner, 2002) and several other models, the neuron is constantly decaying to a *rest potential v*_*rest*_. If a neuron integrates enough input and reaches its *threshold voltage v*_*thr*_, it emits a spike which travels to downstream neurons via synapses, its *post-synaptic* effect modulated by synaptic strengths, and its voltage is reset to some value *v*_*reset*_. Synapses between neurons can also have their own dynamics, which are modified by prescribed learning rules or external reward signals.

Several software packages for the discrete-time simulation of SNNs exist, with varying levels of biological realism and support for hardware platforms. Many such solutions, however, were not developed to target ML applications, and often feature abstruse syntax resulting in steep learning curves for new users. Moreover, packages with a large degree of biological realism may not be appropriate for problems in ML, since they are computationally expensive to simulate and may require a large degree of hyper-parameter tuning. Real-time hardware implementations of SNNs exist as well, but cannot support the rapid prototyping that some software solutions can.

Motivated by the foregoing shortcomings, we present the BindsNET spiking neural networks library, which is developed on top of the popular PyTorch deep learning library (Paszke et al., 2017). At its core, the software allows users to build, train, and evaluate SNNs composed of groups of neurons and their connections. The learning of connection weights is supported by various algorithms from the biological learning literature (Hebb, 1949; Markram et al., 1997). A separate module provides an interface to the OpenAI gym (Brockman et al., 2016) reinforcement learning (RL) environments library from BindsNET. A Pipeline object is used to streamline the interaction between spiking networks and RL environments, removing many of the messy details from the purview of the experimenter. Still other modules provide functions such as loading of ML datasets, encoding of raw data into spike train network inputs, plotting of network state variables and outputs, and evaluation of SNN as ML models.

The paper is structured as follows: we begin in section 2 with an assessment of the existing SNN simulation software and hardware implementations. In section 3, the BindsNET library is described in details, emphasizing the motivation of creating each software module, describing their functionalities, and they way the inter-operate when solving a specific task. Code snippets and simple case studies are introduced in section 4 to demonstrate the breadth of possible BindsNET applications. Desirable directions and features of future developments are listed in 5, while potential research impacts are assessed in section 6.

## 2. Review of SNN Software Packages

### 2.1. Objectives of SNN Simulations

In the last two decades, neural networks have become increasingly prominent in machine learning and artificial intelligence research, leading to a proliferation of efficient software packages for their training, evaluation, and deployment. On the other hand, the simulation of the “third generation” of neural networks (SNNs) has not been able to reach its full potential, due in part to their inherent complexity and computational requirements. However, spiking neurons excel at remembering a short-term history of their activation and feature efficient binary communication with other neurons, a useful feature in reducing energy requirements on neuromorphic hardware. Spiking neurons exhibit more properties from their biological counterpart than the computing units utilized by deep neural networks, which may constitute an important advantage in terms of practical computational power or ML performance.

Researchers that want to conduct experiments with networks of spiking neurons for ML purposes have a number of options for SNN simulation software. Many frameworks exist, but each is tailored toward specific application domains. In this section, we describe the existing relevant software libraries and the challenges associated with each, and contrast these with the strengths of our package.

We believe that the chosen simulation framework must be easy to develop in, debug, and run, and, most importantly, support the level of biological complexity desired by its users. We express a preference to maintain consistency in development by using a single programming language, and for it to be affordable or an open source project. We describe whether and how these aspects are realized in each competing solution.

### 2.2. Comparison of State-of-Art Simulation Packages

Many spiking neural network frameworks exist, each with a unique set of use cases. Some focus on the biologically realistic simulation of neurons, while others on high-level spiking network functionality. To build a network to run even the simplest machine learning experiment, one will face multiple difficult design choices: Which biological properties should the neurons and the network have? e.g., how many GABAergic neurons or NMDA/AMPA receptors should be used, or what form of synaptic dynamics? Many such options exist, some of which may or may not have a significant impact on the performance of an ML system.

Several prominent SNN simulation packages are compared in Table 1. For example, NEST (Gewaltig and Diesmann, 2007), BRIAN (Stimberg et al., 2014), and ANNarchy (Vitay et al., 2015) focus on accurate biological simulation from sub-cellular components and biochemical reactions, to complex models of single neurons, all up to the network level. Other popular biologically realistic platforms are NEURON (Carnevale and Hines, 2006), Genesis (Cornelis et al., 2012). These simulation platforms target the neuro-biophysics community and neuroscientists that wish to simulate multicompartment neuron models, in which each compartment is a different part of the neuron with different functionalities, morphological details, and shape. These packages are able to simulate large SNNs on various types of systems, from laptops all the way up to HPC systems. However, each simulated component must be *homogeneous*, meaning that it must be built with a single type of neuron and a single type of synapse. If a researcher wants to simulate multiple types of neurons utilizing various synapse types, it may be difficult in these frameworks. For a more detailed comparison of development time, model performance, and varieties of models of neurons available in these libraries see (Tikidji-Hamburyan et al., 2017).

**Table 1:** Comparison between spiking neural network simulation libraries.

**Simulator**	**Affiliation**	**Open source**	**Simulation**	**OpenMP**	**GPU**	**Programming languages**
ANNarchy	Chemnitz University	Yes	Clock-	Yes	Yes	C++
	Germany		driven			with Python interface
(Py)NEST	University of Freiburg	Yes	Hybrid	Yes	No	C++
	Germany					with Python interface
CARLsim	University of California	Yes	Clock-	Yes	Yes	C++
	Irvine, CA, US		driven			with PyNN support
NeMo	Imperial College	Yes	Clock-	Yes	Yes	C++
	London, UK		driven			with Python & PyNN support
PyNN	Open Community	Yes	Various	Yes	Yes	Python
						Interface only
Nengo AI	University of Waterloo	Yes	Clock-	Partially	Yes	C++
	Canada		driven			with Python wrapper
SpiNNaker	Manchester University	Yes	Event-	No	No	C++ with
	UK		driven			PyNN & sPyNNaker support
Brian 2	Ecole Normale Superieure	Yes	Clock-	Yes	No	C++
	Paris, France		driven			with Python wrapper
Brain2GeNN	University of Sussex	Yes	Clock-	Yes	Yes	C++
(GeNN)	UK		driven			with Python wrapper
NeuCube	Auckland University	No	?	?	?	MATLAB
	New Zealand					
BindsNET	University Massachusetts	Yes	Clock-	Yes	Yes	C++
	Amherst, US		driven			with Python wrapper

A major benefit of the BRIAN, ANNarchy, NEST, and NEURON packages is that, besides the built-in modules for neuron and connection objects, the programmer is able to specify the dynamics of neurons and connections using differential equations. This eliminates the need to manually specify the dynamic properties of each new neuron or connection object in code. The equations are compiled into fast C++ code in the case of ANNarchy, vectorised and linear algebraic operations using NumPy and Basic Linear Algebra Subprograms (BLAS) in the case of BRIAN2, and to a mix of Python and native C-like language (hoc) (Hines et al., 2009) which are responsible for SNN simulation in the case of NEURON. In addition, in the NEST package, the programmer can combine pre-configured objects (which accepts arguments) to create SNNs. In all of these libraries, significant changes to the operation of the network components requires modification of the underlying code, a difficult task which gets in the way of fast network prototyping and breaks the continuity of the programming. At this time, BindsNET does not support the solution of arbitrary differential equations describing neural dynamics, rather, for simplicity, several popular neuron types are provided for the user to chose from.

Frameworks such as NeuCube (Kasabov, 2014) and Nengo (Bekolay et al., 2014) focus on high-level behaviors of spiking neural networks and may be used for machine learning experimentation. NeuCube supports rate coding-based spiking networks, and Nengo supports simulation at the level of spikes, firing rates, or high-level, abstract neural behavior. NeuCube attempts to map spatiotemporal input data into three-dimensional SNN architectures; however, it is not an open source project, and therefore is somewhat restricted in scope and usability. Nengo is often used to simulate high-level functionality of brains or brain regions, as a cognitive modeling toolbox implementing the Neural Engineering Framework (Stewart, 2012) rather than a machine learning framework. Nengo is an open source project, written in Python, and supports a Tensorflow (Abadi et al., 2015) backend to improve simulation speed and exploit some limited ML functionality. It also has options for deploying neural models on dedicated hardware platforms; e.g., SpiNNaker (Plana et al., 2011). CARLsim (Beyeler et al., 2015) and NeMo (Fidjeland et al., 2009) also focus on the high-level aspects of SNNs and are thus good candidates for applications in machine learning. Both allow the simulation of large spiking networks built with Izhikevich neurons (Izhikevich, 2003) with realistic synaptic dynamics as their fundamental computational unit, and support accelerated computation with GPU hardware. Like the frameworks before, low-level simulation code is written in C++ for efficiency, but programmers can interact with them with a simulator-independent PyNN Python library (Davison et al., 2008), or in MATLAB or Java.

The GeNN (GPU-enhanced neuronal networks) library Yavuz et al. (2016) is an environment that enables simulation of SNNs on CPUs or NVIDIA GPUs via code generation technology. Networks are defined in a C-style API, and the code for simulating them (on CPU or GPU) are automatically generated by GeNN. The recent BRIAN2genn package Stimberg et al. (2018) (in beta release) can be used to convert network models written in BRIAN2 to run on NVIDIA GPUs using the GeNN library, by invoking BRIAN2's set_device() function to execute code in an external framework. Although this platform targets both CPUs and GPUs (a central feature of the BindsNET library), it requires an (often costly) intermediate code generation step between network prototyping and deployment (see Figure 11 for an illustration of this issue). It is also difficult to intervene on the generated code when running; e.g., clamping synapses if certain criteria are met, or changing learning rates as the simulation progresses.

Many of the above packages are written in more than one programming language: the core functionality is implemented in a lower-level language (e.g., C++) to achieve good performance with low overhead, and the code exposed to the user of the package is written in a higher-level language (e.g., Python or MATLAB) to enable fast prototyping. If such frameworks are not tailored to the needs of a user, have steep learning curves, or aren't flexible enough to create a desired model, the user may have to program in both high- and low-level languages to make changes to the required internal components. The authors have encountered this difficulty with the BRIAN2 library in particular, since certain segments of simulation functionality is regulated to generated code, which is difficult or impossible to modify while, for example, training a SNN for a machine learning task. This issue is likely to appear in similar software frameworks; e.g., GeNN and ANNarchy.

BindsNET relies on PyTorch for its matrix computations in order to perform efficient simulation of spiking neural networks. Without changing the details of the mathematical operations, BindsNET can in principle be connected to various hardwares, e.g., FPGA, ASIC, DSP, or ARM, to execute the simulations. One may design an API to compile spiking networks created in BindsNET to run on designated hardware instead of using PyTorch as the simulation workhorse. In this way, BindsNET can be seen as a bridge between the software and hardware domains, enabling researchers to rapidly test software prototypes on CPUs or GPUs, and eventually deploy the simulation to fast, energy efficient dedicated hardware. At the moment, no such API exists, but may be added in a future release of the library.

## 3. Package structure

**Figure 1:**
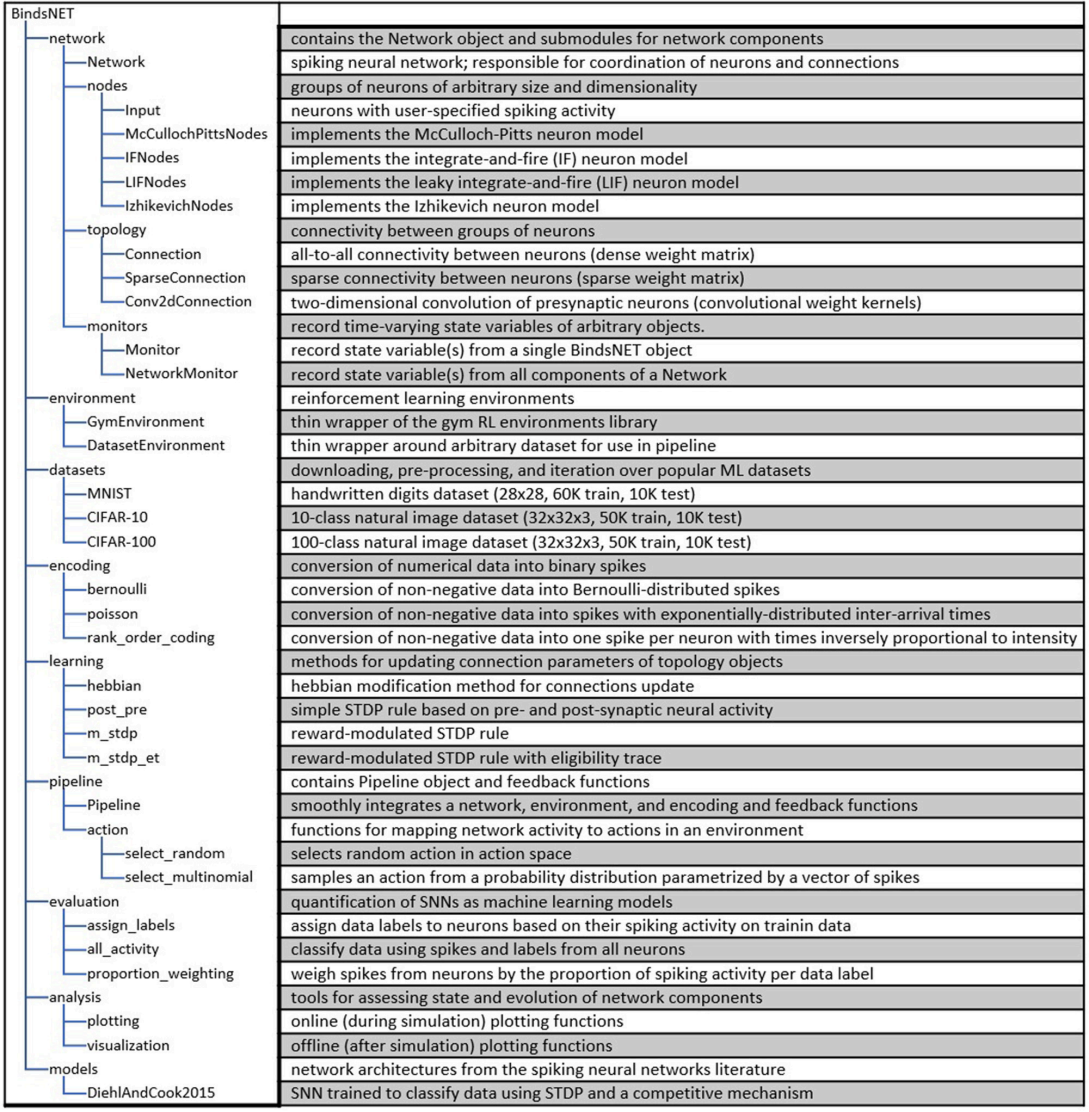
Depiction of the BindsNET directory structure and description of major software modules.

A summary of all the software modules of the BindsNET package is included in Figure 1.

Many BindsNET objects use the torch.Tensor data structure for computation; e.g., all objects supporting the Nodes interface use Tensors to store and update state variables such as spike occurrences or voltages. The Tensor object is a multi-dimensional matrix containing elements of a single data type; e.g., integers or floating points numbers with 8, 16, 32, or 64 bits of precision. They can be easily moved between devices with calls to Tensor.cpu() or Tensor.cuda(), and can target GPU devices by default with the statement torch.set_default_tensor_type('torch.cuda.FloatTensor').

### 3.1. SNN Simulation

BindsNET provides a Network object (in the network module) which is responsible for the coordination of one or many Nodes and Connections objects, and supports the use of Monitors for recording the state variables of these components. A time step parameter dt is the sole (optional) argument to the Network constructor, which controls the temporal resolution of simulation. The run(inpts, time) function implements synchronous updates (for a number of time steps $\frac{\text{time}}{\text{dt}}$) of all network components. This function calls get_inputs() to calculate pre-synaptic inputs to all Nodes instances (alongside user-defined inputs in inpts) as a subroutine. A reset_() method invokes resetting functionality of all network components, namely for resetting state variables back to default values. Saving and loading of networks to and from disk is implemented, permitting re-use of trained connection weights or other parameters.

The Nodes abstract base class in the nodes module specifies the abstract functions step(inpts, dt) and reset_(). The first is called by the run() function of a Network instance to carry out a single time step's update, and the second resets spikes, voltages, and any other recorded state variables to default values. Implementations of the Nodes class include Input (neurons with user-specified or fixed spikes) McCullochPittsNodes (McCulloch-Pitts neurons), IFNodes (integrate-and-fire neurons), LIFNodes (leaky integrate-and-fire neurons), and IzhikevichNodes (Izhikevich neurons). Other neurons or neuron-like computing elements may be implemented by extending the Nodes abstract class. Many Nodes object support optional arguments for customizing neural attributes such as threshold, reset, and resting potential, refractory period, membrane time constant, and more. It should be noted that some Nodes objects' behavior does not depend on the dt parameters; for example, the McCullochPittsNodes object has no memory of previous time steps (stateless), and yet it may still be embedded in a SNN simulation.

The topology module is used to specify interactions between Nodes instances, the most generic of which is implemented in the Connection object. The Connection is aware of *source* (pre-synaptic) and *target* (post-synaptic) Nodes, as well as a matrix of weights w of connections strengths. By default, connections do not implement any learning of connection weights, but do so through the inclusion of an update_rule argument. Several canonical learning rules from the biological learning literature are implemented in the learning module, including Hebbian learning (Hebbian), a variant of spike-timing-dependent plasticity (STDP) (PostPre), and less well-known methods such as reward-modulated STDP (MSTDP). The optional argument norm to the Connection specifies a desired sum of weights per target neuron, which is enforced by the parent Network during each call of run(). A SparseConnection object is available for specifying connections where certain weights are fixed to zero; however, this does not yet available for learning functionality due to a lack of adequate support for sparse Tensor in the PyTorch library. The Conv2dConnection object implements a two-dimensional convolution operation (using PyTorch's torch.nn.conv2d function) and supports all update rules from the learning module. The LocallyConnectedConnection implements a two-dimensional convolutional layer without shared weights; i.e., each input region is associated with a different set of filter weights (Bruna et al., 2013; Saunders et al., 2018).

### 3.2. Machine and Reinforcement Learning

BindsNET is being developed with machine and reinforcement learning applications in mind. At the core of these efforts is the learning module, which contains functions which can be attached to Connection objects to modify them during SNN simulation. By default, connections are instantiated with no learning rule. The Hebbian rule (“fire together, wire together”) symmetrically strengthens weights when pre- and post-synpatic spikes occur temporally close together, and the PostPre rule implements a simple form of STDP in which weights are increased or decreased according to the relative timing of pre- and post-synaptic spikes, with user-specified (possibly asymmetric) learning rates. The reward-modulated STDP (MSTDP) and reward-modulated STDP with eligibility trace (MSTDPET) rules of Florian (2007) are also implemented for use in basic reinforcement learning experiments. In general, any learning rule can be used with any connection types and other network components, but it is up to the researcher to choose the right method for their experiment.

The datasets module provides a means to download, pre-process, and iterate over machine learning datasets. For example, the MNIST object provides this functionality for the MNIST handwritten digits dataset. Several other datasets are supported besides, including CIFAR-10, CIFAR-100, (Krizhevsky and Hinton, 2009) and Spoken MNIST. The samples from a dataset can be encoded into spike trains using the encoding module, currently supporting several functions for creating spike trains from non-negative data based on different statistical distributions and biologically inspired transformations of stimuli. Encoding functions include poisson(), which converts data representing firing rates into Poisson spike trains with said firing rates, and rank_order(), which converts data into single spikes per neuron temporally ordered by the intensity of the input data (Thorpe and Gautrais, 1998). Spikes may be used as input to SNNs, or even to other ML systems. A submodule preprocess of datasets allows the user to apply various pre-processing techiques to raw data; e.g., cropping, subsampling, binarizing, and more.

The environment module provides an interface into which a SNN, considered as a reinforcement learning agent, can take input from and enact actions in a reinforcement learning environment. The GymEnvironments object comprises of a generic wrapper for gym (Brockman et al., 2016) RL environments and calls its reset(), step(action), close(), and render() functionality, while providing a default pre-processing function preprocess() for observations from each environment. The step(action) function takes an action in the gym environment, which returns an observation, reward value, an indication of whether the episode has finished, and a dictionary of (name, value) pairs containing additional information. Another object, DatasetEnvironment, provides a generic wrapper around objects from the datasets module, allowing these to be used as a component in a Pipeline instance (see section 3.3). The environment.action module provides methods for mapping one or more network layers' spikes to actions in the environment; e.g., select_multinomial() treats a (normalized) vector of spikes as a probability distribution from which to sample an action for the environment's similarly-sized action space.

Simple methods for the evaluation of SNNs as machine learning models are implemented in the evaluation module. In the context of unsupervised learning, the assign_labels() function assigns data labels to neurons corresponding to the class of data on which they spike most during network training (Diehl and Cook, 2015). These labels are to classify new data using methods like all_activity() and proportion_weighting() (Hazan et al., 2018). We have recently added logreg_fit and logreg_predict methods for fitting and predicting on categorical data with the logistic regression implementation borrowed from the scikit-learn library (Pedregosa et al., 2011). We plan to add additional “read-out” methods in the near future, such as *k*-nearest neighbor (KNN) and support vector machines (SVMs).

A collection of network architectures is defined in the models module. For example, the network structure of Diehl and Cook (2015) is implemented by the DiehlAndCook2015 object, which supports arguments such as n_neurons, excite, inhib, etc. with reasonable default values.

### 3.3. The Pipeline Object

As an additional effort to ease prototyping of machine learning systems comprising spiking neural networks, we have provided the Pipeline object to compose an environment, network, an encoding of environment observations, and a mapping from network activity to the environment's action space. The Pipeline also provides optional arguments for visualization of the environment and network state variables during network operation, skipping or recording observations on a regular basis, the length of the simulation per observation (defaults to 1 time step), and more. The main action of the pipeline can be explained as a four-step, recurring process, implemented in the pipeline step() function:

1. An action is selected based on the activity of one or more of the network's layers during the last one or more time steps
2. This action is used as input to the environment's step() function, which returns a new observation, a scalar reward, whether the simulation has finished, and any additional information particular to the environment
3. The observation returned from the environment is converted into spike trains according to the user-specified encoding function (either custom or from the encoding module) and request simulation time
4. The spike train-encoded observation is used as input to the network.

Alongside the required arguments for the Pipeline object (network, environment, encoding, and action), there are a few keyword arguments that are supported, such as history and delta. The history_length argument indicates that a number of sequential observations are to maintained in order to calculate differences between current observations and those stored in the history data structure. This implies that only new information in the environment's observation space is delivered as input to the network on each time step. The delta argument (default 1) specifies an interval at which observations are stored in history. This may be useful if observations don't change much between consecutive steps; then, we should wait some delta time steps between taking observations to expect significant differences. As an example, combining history_length = 4 and delta = 3 will store observations {0, 3, 6, 9}, {3, 6, 9, 12}, {6, 9, 12, 15}, etc. A few other keyword arguments for handling console output, plotting, and more exist and are detailed in the Pipeline object documentation.

**Figure 2:**
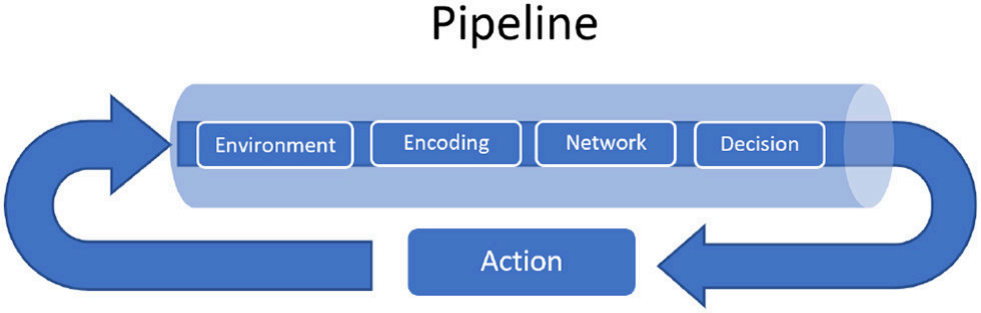
A functional diagram of the Pipeline object. The four-step process involves an encoding function, network computation, converting network outputs into actions in an environment's action space, and a simulation step of the environment. An encoding function converts non-spiking observations from the environment into spike inputs to the network, and a action function maps network spiking activity into a non-spiking quantity: an action, fed back into the environment, where the procedure begins anew. Other modules come into play in various supporting roles: the network may use a learning method to update connection weights, or the environment may simply be a thin wrapper around a dataset (in which case there is no feedback), and it may be desirable to plot network state variables during the reinforcement learning loop.

A functional diagram of the Pipeline object is depicted in Figure 2.

### 3.4. Visualization

BindsNET contains useful visualization tools that provide information during or after network or environment simulation. Several generic plotting functions are implemented in the analysis.plotting module; e.g., plot_spikes() and plot_voltages() create and update plots dynamically instead of recreating figures at every time step. These functions are able to display spikes and voltages with a single call. Other functions include plot_weights() (displays connection weights), plot_input() (displays raw input data), and plot_performance() (displays time series of performance metric). Other visualization libraries in the Python ecosystem such as matplotlib can be used to plot network state variables or other data as users of BindsNET may require for more complicated use cases not covered by the plotting module.

The analysis.visualization module contains additional plotting functionality for network state variables after simulation has finished. These tools allow experimenters to analyze learned weights or spike outputs, or to summarize long-term behaviors of their SNN models. For example, the weights_movie() function creates an animation of a Connection's weight matrix from a sequence of its values, enabling the visualization of the trajectory of connection weight updates.

### 3.5. Adding New BindsNET Features

To extend BindsNET, one can extend certain abstract objects found in the package with the desired functionality. In the following, we discuss how new neuron models, connection types, and learning rules can be custom-defined by users and developers of BindsNET. Other BindsNET objects (e.g., Monitors, Datasets, etc.) can be defined in a similar fashion.

#### 3.5.1. Neuron Models

The abstract class Nodes implements functionality that is common to all neuron types. It defines the abstract functions step() and reset_(), which one can choose to override in child classes, or to One can define a new Nodes object by writing a class of the form:


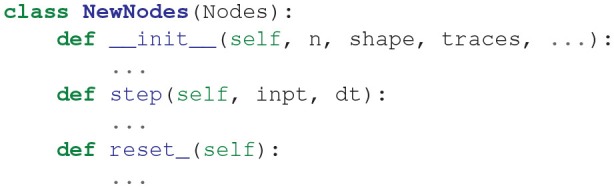


All three functions typically call the similarly-named Nodes abstract class functions, but it is possible to completely re-define the functions as needed. The abstract base class AbstractInput is also available for defining node types with user-defined inputs (e.g., for simulating constant current injection with the RealInput object).

At present, BindsNET does not automatically solve state variable dynamics equations (as does, for example, the BRIAN simulator Goodman and Brette, 2009); instead, the user must define the neuron difference equation themselves in the body of the step() function. We implement Euler integration as part of our emphasis on efficient computation. Automatic solution of dynamics equations may be added in a future release of BindsNET.

#### 3.5.2. Connection Types

The class AbstractConnection implements functionality common to all connection objects. It defines the abstract methods compute(s), update(dt), normalize(), and reset_(). Users of BindsNET can define their own connection types by creating a class that inherits from AbstractConnection. To define a new connection object, one must write a class of the form:


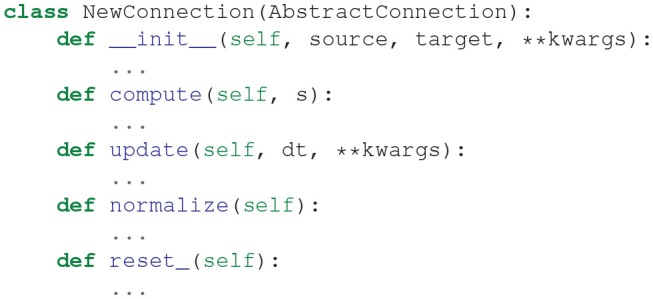


#### 3.5.3. Learning Rules

The abstract class LearningRule defines functions common to all learning rules. It defines the abstract method update(dt), used to update a connection's synapse strengths in some fashion. Typically, this method makes use of pre- and post-synaptic neuron spikes and / or spike traces in order to calculate some local learning rule; e.g., PostPre STDP. However, users of BindsNET may want to construct learning rules than depend on non-local information; e.g., the MSTDP and MSTDPET rules require a reward keyword argument to modulate the sign and strength of synapse weight updates. To define a new learning rule, one can write a class as follows:


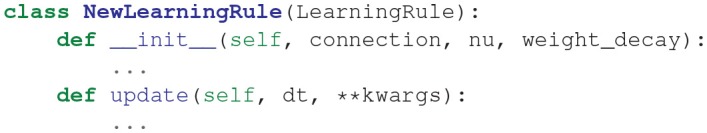


## 4. Examples of Using BindsNET to Solve Machine Learning Tasks

We present some simple example scripts to give an impression of how BindsNET can be used to build spiking neural networks implementing machine learning functionality. BindsNET is built with the concept of encapsulation of functionality to make it faster and easier for generalization and prototyping. Note in the examples below the compactness of the scripts: fewer lines of code are needed to create a model, load a dataset, specify their interaction in terms of a pipeline, and run a training loop. Of course, these commands rely on many lines of underlying code, but the user no longer has to implement them for each experimental script. If changes in the available parameters are not enough, the experimenter can intervene by making changes in the underlying code in the model without changing language or environment, thus preserving the continuity of the coding environment.

### 4.1. Unsupervised Learning

**Figure 3:**
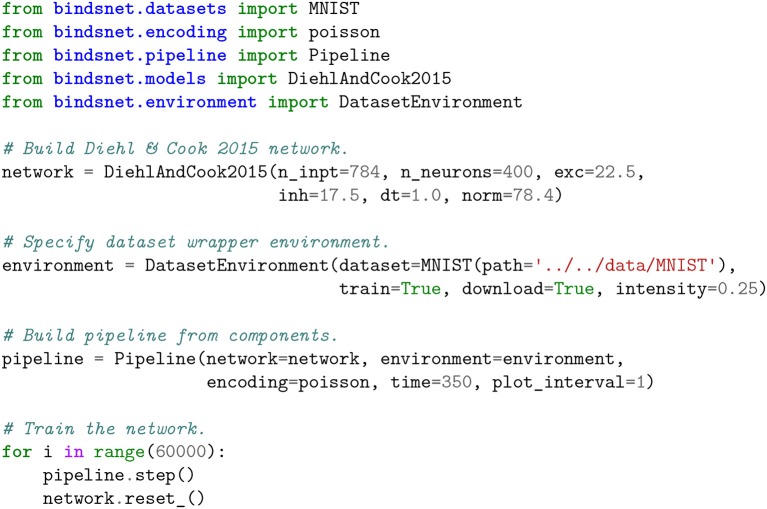
Accompanying plots to the unsupervised training of the DiehlAndCook2015 spiking neural network architecture. The network is able to learn prototypical examples of images from the training set, and on a test images, the excitatory neuron with the most similar filter should fire the most. This network structure is able to achieve 95% accuracy on the MNIST digits (Diehl and Cook, 2015; Hazan et al., 2018). **(A)** Raw input and “reconstructed” input, computed by summing Poisson-distributed spike trains over the time dimension. **(B)** Spikes from the excitatory and inhibitory layers of the DiehlAndCook2015 model. **(C)** Voltages from the excitatory and inhibitory layers of the DiehlAndCook2015 model. **(D)** Reshaped 2D label assignments of excitatory neurons, assigned based on activity on examples from the training data. **(E)** Reshaped 2D connection weights from input to excitatory layers. The network is able to learn distinct prototypical examples from the dataset, corresponding to the categories in the data.

**Figure 4:**
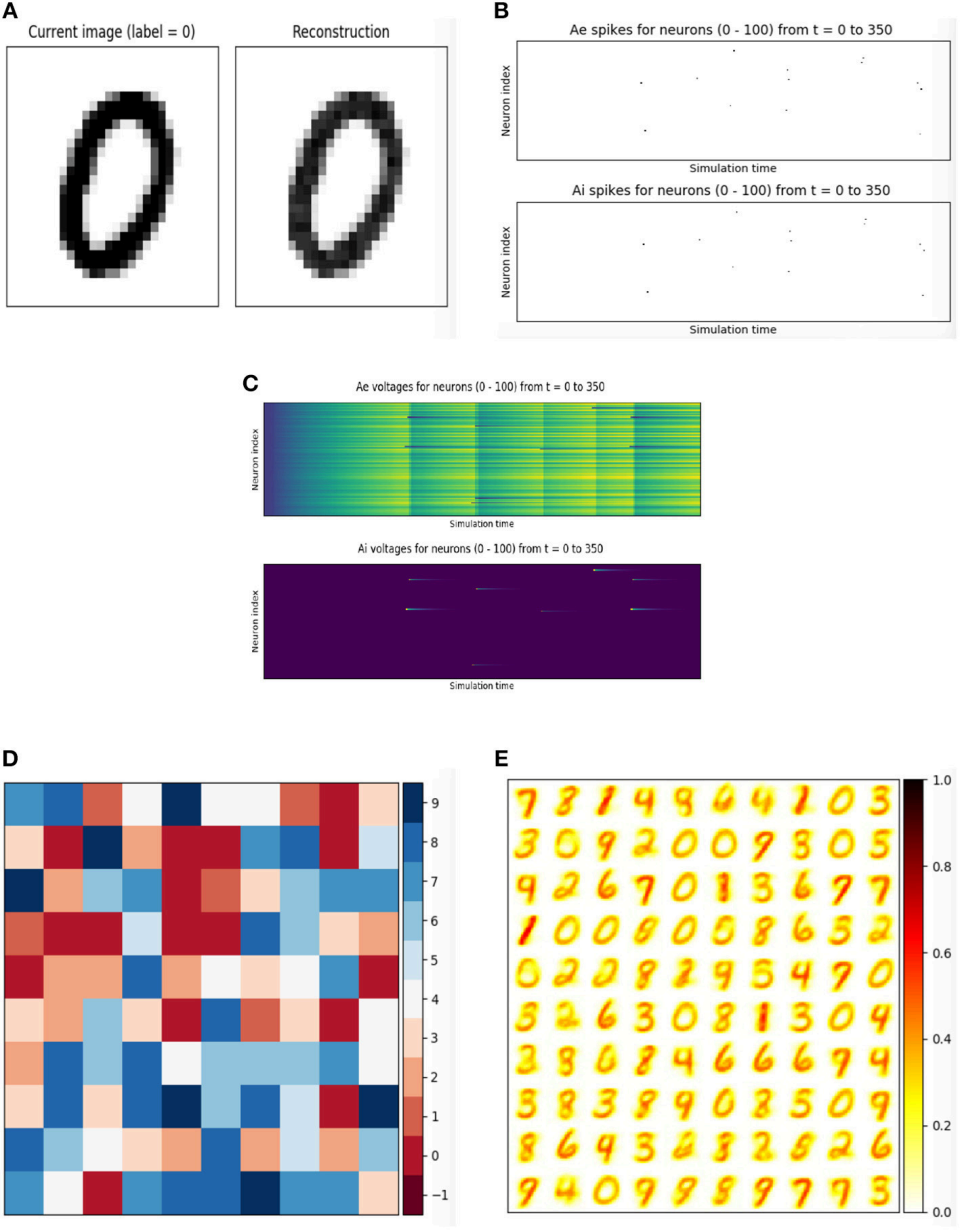
Unsupervised learning of the MNIST handwritten digits in BindsNET. The DiehlAndCook2015 model implements a simple spike timing-dependent plasticity rule between input and excitatory neuron populations as well as a competitive inhibition mechanism to learn prototypical digit filters from raw data. The DatasetEnvironment wraps the MNIST dataset object so it may be used as a component in the Pipeline. The network is trained on one pass through the 60K-example training data for 350ms each, with state variables (voltages and spikes) reset after each example.

The DiehlAndCook2015 object in the models module implements a slightly simplified version of the network architecture discussed in Diehl and Cook (2015). A minimal working example of training a spiking neural network to learn, without labels, a representation of the MNIST digit dataset is given in Figure 3, and state variable-monitoring plots are depicted in Figure 4. The Pipeline object is used to hide the messy details of the coordination between the dataset, encoding function, and network instance. Code for additional plots or console output may be added to the training loop for monitoring purposes as needed.

The main goal of the present paper is to introduce the BindsNET software framework, while a systematic evaluation of the implementation and comparison with other SNN platforms is the objective of ongoing or future studies. Nevertheless, it is important to show that BindsNET measures up to its peers. To illustrate the performance of BindsNET, here we introduce some preliminary results; further details are given in Saunders et al. (2018) and Hazan et al. (2018). In the case of MNIST dataset, BindsNET's classification performance reaches 95%, which is on a par with the BRIAN-based implementations reported in Diehl and Cook (2015). Moreover, BindsNET's flexible platform allowed extensive exploration of learning rules and hyper-parameters, and we have shown that our approach can reach or exceed BRIAN's accuracy with smaller SNNs. Moreover, as training progresses, the accuracy of our approach using BindsNET increases rapidly at the early stage of learning, using much less examples than alternative methods (Hazan et al., 2018). Again, in the present work we do not aim at a systematic evaluation of the solutions based on BindsNET, but the initial results are promising, and extensive work is in progress.

### 4.2. Supervised Learning

**Figure 5:**
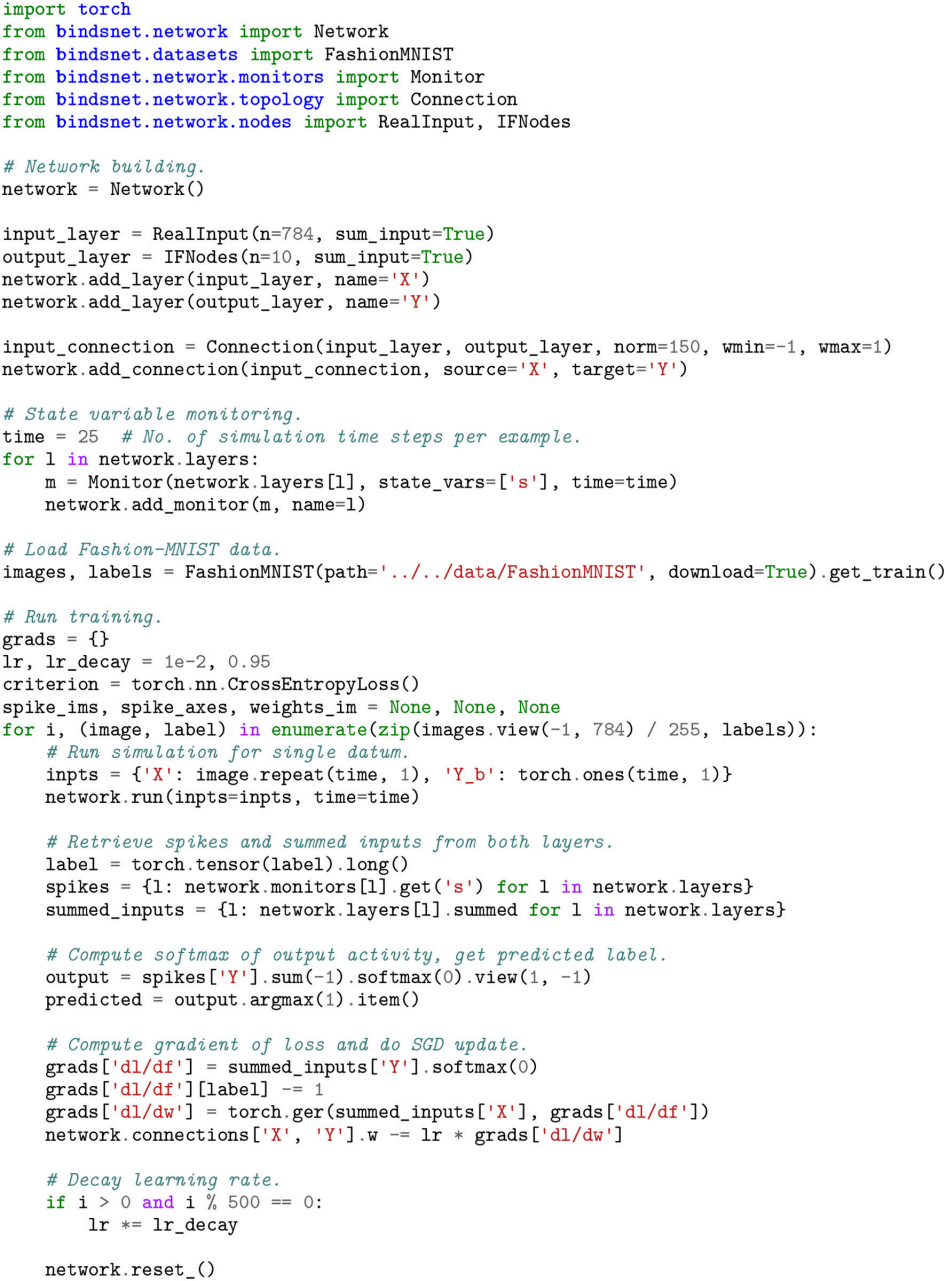
A two-layer spiking neural network (a RealNodes object connected all-to-all with a IFNodes object) is trained with an approximated stochastic gradient descent algorithm using the Fashion-MNIST image dataset. The back-propagation algorithm operates on the summed_inputs to the groups of Nodes, while predictions are made based on the output layer's spiking activity.

**Figure 6:**
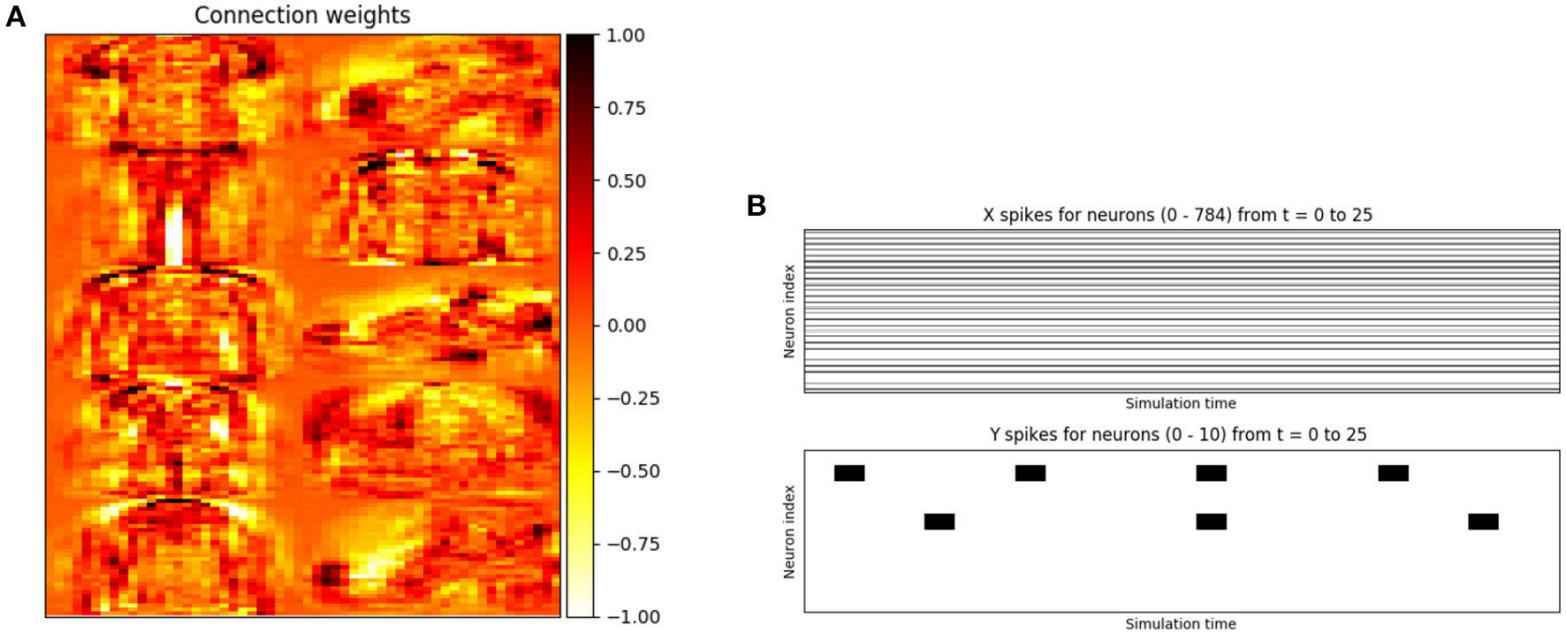
Accompanying plots for the supervised training of a simple two-layer spiking neural network on the Fashion-MNIST dataset. The set of 10 28 × 28 tiled weights shown in (a) each correspond to a different class of Fashion-MNIST data. The plot of the input neurons' activity in (b) is simply the scaled input data, constant over the simulation length. This network architecture trained with stochastic gradient descent (SGD) achieves 85% test accuracy on this dataset. **(A)** Weights from the supervised spiking neural network trained on the Fashion-MNIST dataset. Each 28 × 28 region corresponds to the filter responsible for detecting a unique category of data. One can make out the profile of objects depicted in the filters; e.g., shirts, sneakers, and trousers. **(B)** Real-valued input activity and spikes from the input and output layers of the two-layer network, respectively.

We used a simple two-layer spiking neural network to implement supervised learning of the Fashion-MNIST image dataset (Xiao et al., 2017). An minimal example of training a spiking network to classify the data is given in Figure 5, with plotting outputs depicted in Figure 6. A layer of 100 excitatory neurons is split into 10 groups of size 10, one for each category. On each input example, we observe the label of the data and clamp a randomly selected excitatory neuron from its group to spike on every time step. This forces the neuron to adjust its filter weights toward the shape of current input example.

### 4.3. Reinforcement Learning

**Figure 7:**
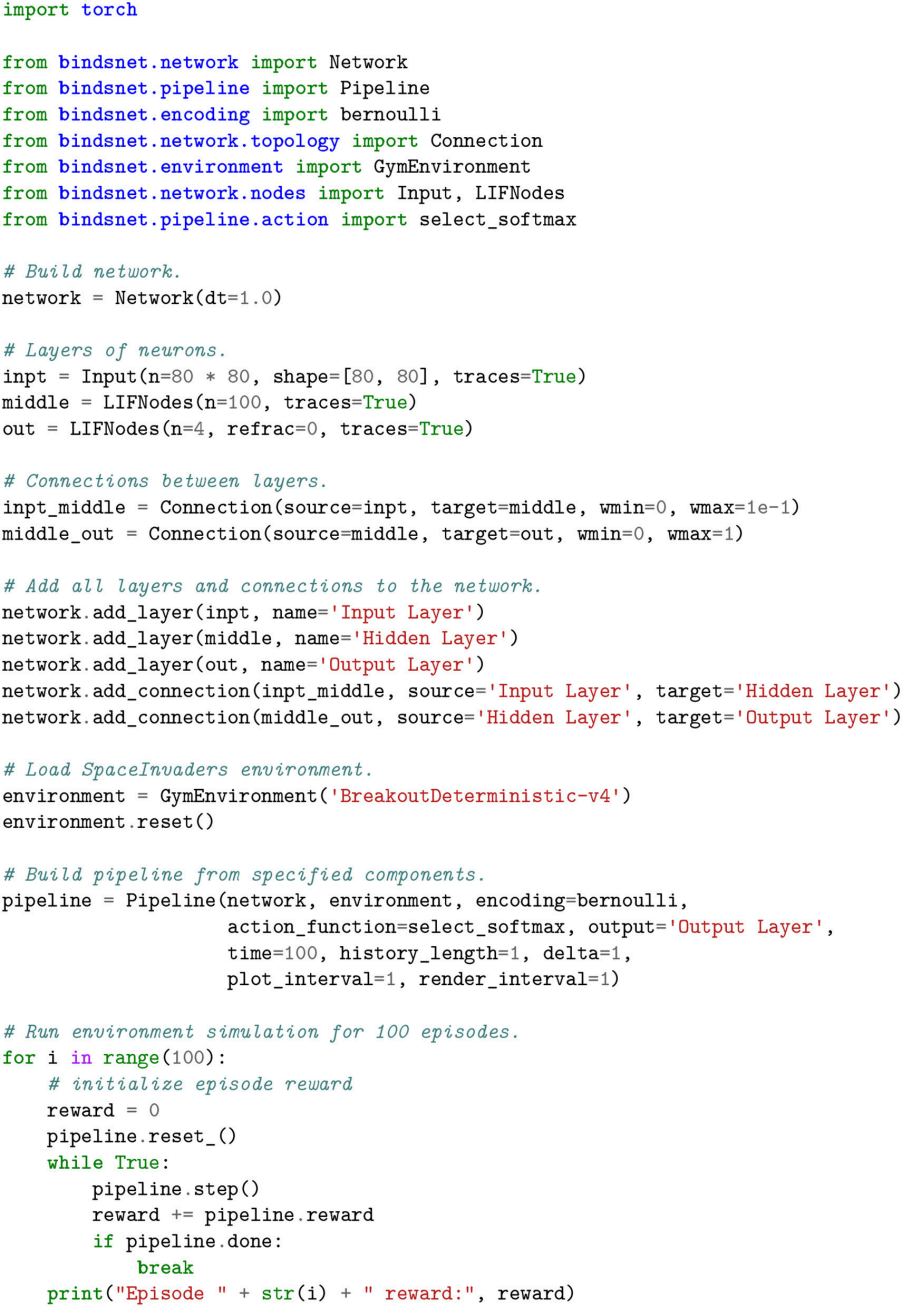
A spiking neural network that accepts input from the BreakoutDeterministic-v4 gym Atari environment. The observations from the environment are downsampled and binarized. The history and delta keyword arguments are used to create difference images before they are converted into Bernoulli-distributed vectors of spikes, one per time step. The output layer of the network has 4 neurons in it, each representing a different action in the Breakout game. An action is selected at each time step using the select_softmax feedback function, which treats the summed spikes over each output layer neuron as a probability distribution over actions.

**Figure 8:**
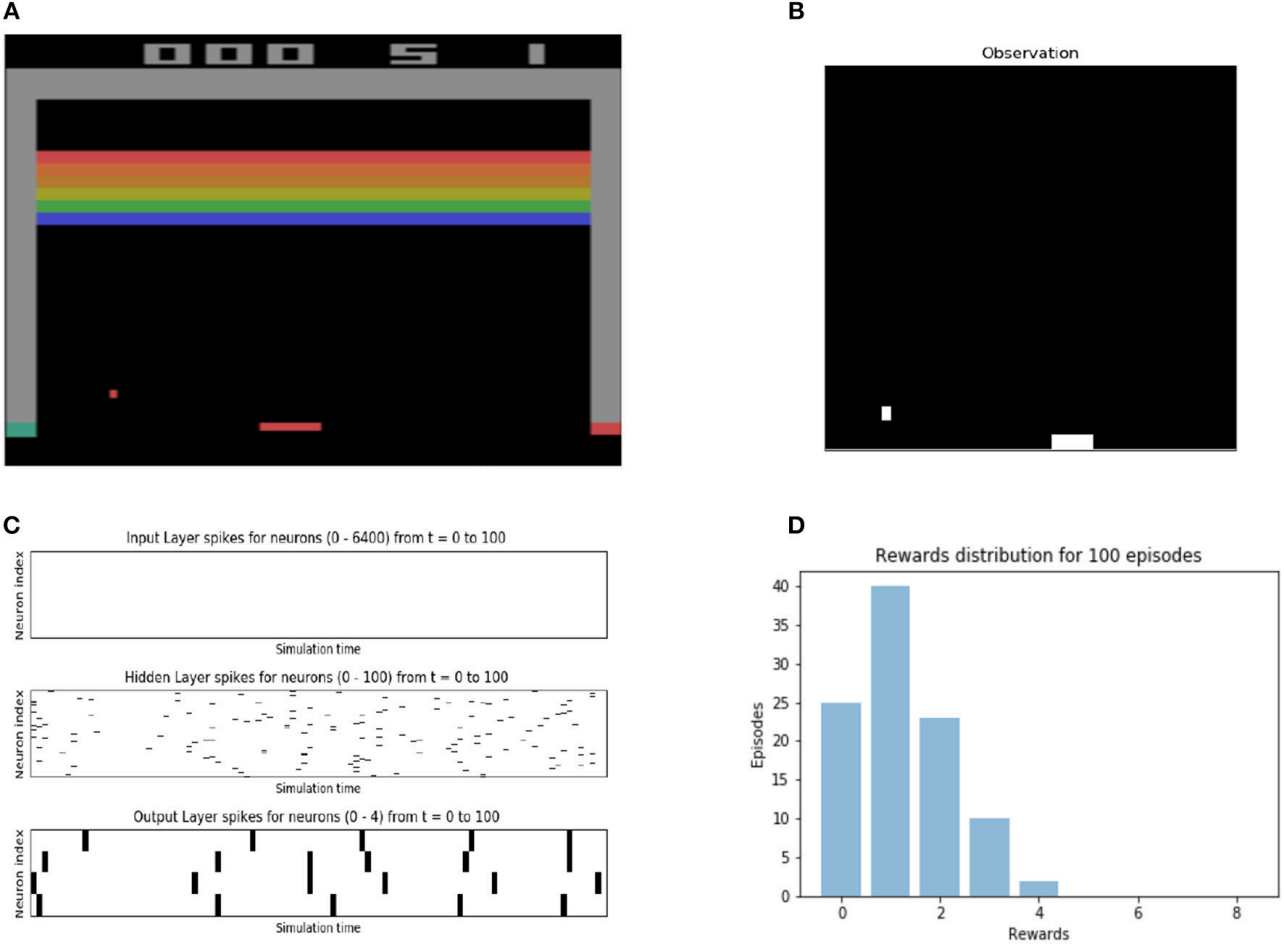
Accompanying plots for a custom spiking neural network's which interacts with the BreakoutDeterministic-v4 reinforcement learning environment. Spikes of all neuron populations are plotted, and the Breakout game is rendered, as well as the downsampled, history- and delta-altered observation, which is presented to the network. The performance of the network on 100 episodes of Breakout is also plotted. (note: The absence of spikes in the Input layer is due to the the large size of the layer and the way matplotlib library handles it. It is not a bug in our code). **(A)** Raw output from the Breakout game, provided by the OpenAI gymrender() method. **(B)** Pre-processed output from breakout game environment used as input to the SNN. **(C)** Spikes from the Input, Hidden, and Output layers of the spiking neural network. **(D)** The reward distribution of the initialized network on 100 episodes of Breakout.

A three layer SNN is built to compute on spikes encoded from Breakout observations. The input layer takes the spike encoding of a 80x80 image which has been downsampled and binarized from the observations from the GymEnvironment. The output layer consists of 4 neurons which correspond to the 4 possible actions for the Breakout game. The result of this computation is spiking activity in the output layer, which are converted into actions in the game's action space by using a softmax function on the sum of the spikes in the output layer. The simulation of both the network and the environment are interleaved and appear to operate in parallel. The SNN combined with the softmax function gives a stochastic policy for the RL environment and the user may apply any reinforcement learning algorithm to modify the parameters of the SNN to change the policy. For a more complete view of the details involved in constructing an SNN and deploying a GymEnvironment instance, see the script depicted in Figure 7 and accompanying displays in Figure 8.

### 4.4. Reservoir Computing

**Figure 9:**
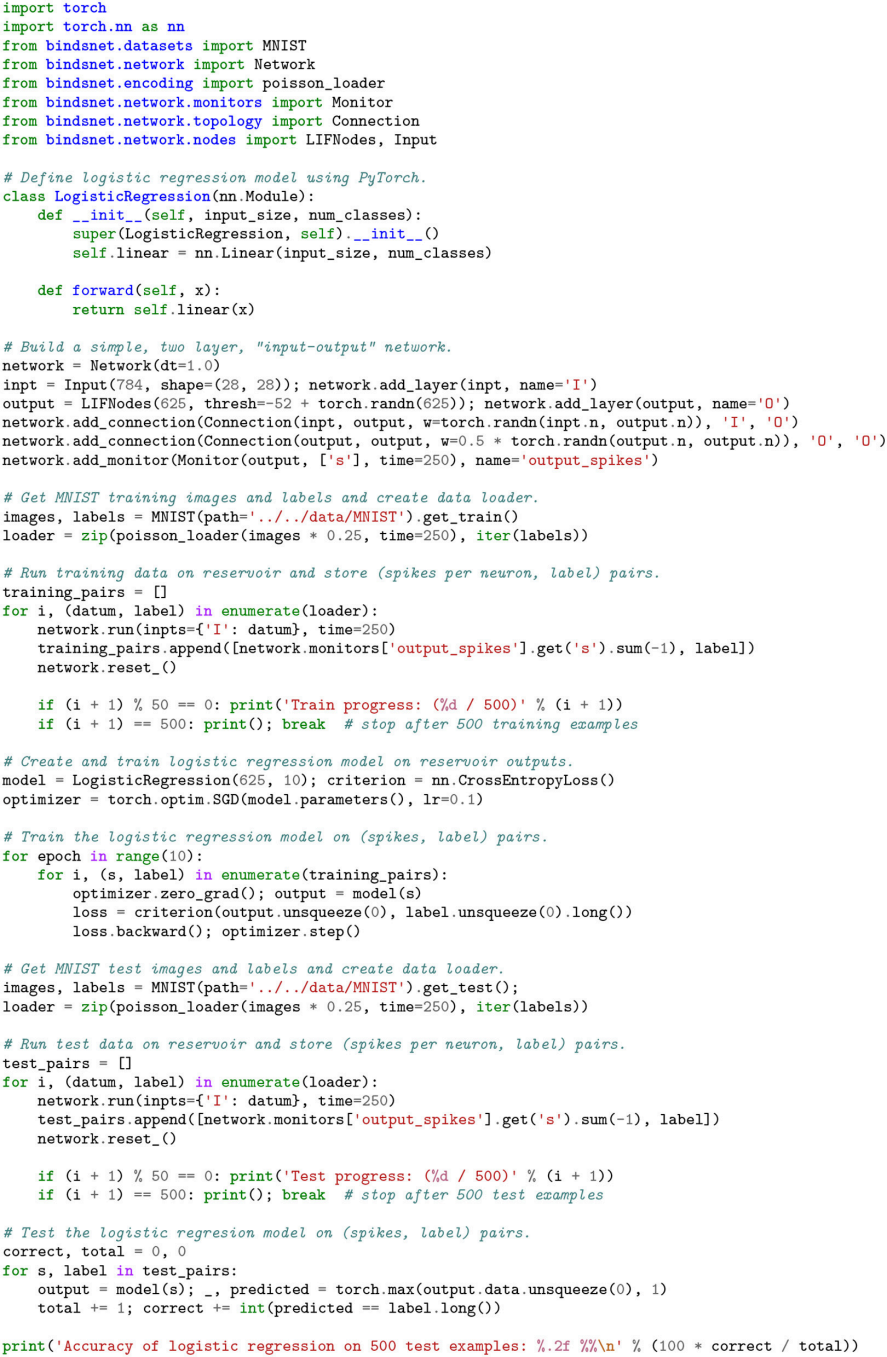
A recurrent neural network built from 625 spiking neurons accepts inputs from the CIFAR-10 natural images dataset. An *input* population is connected all-to-all to an *output* population of LIF neurons with weights draw from the standard normal distribution, which has voltage thresholds drawn from N(-52,1) and is recurrently connected to itself with weights drawn from $N\left(0,\frac{1}{2}\right)$. The reservoir is used to create a high-dimensional, temporal representation of the image data, which is used to train and test a logistic regression model created with PyTorch.

**Figure 10:**
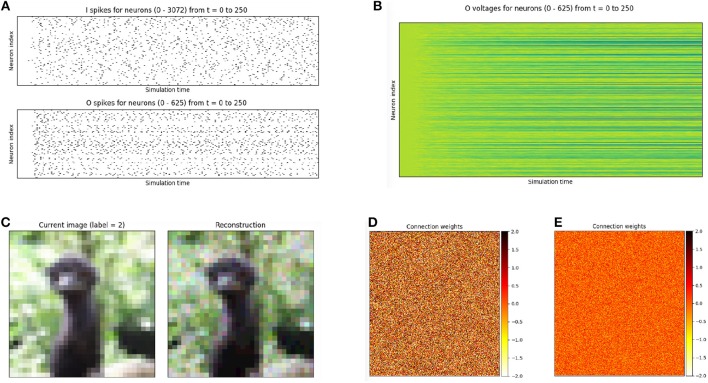
Plots accompanying another reservoir computing example, in which an input population of size equal to the CIFAR-10 data dimensionality is connected to a population of 625 LIF neurons, which is recurrently connected to itself. **(A)** Spikes recorded from the input and output layers of the two layer reservoir network. **(B)** Voltages recorded from the output of the two layer reservoir network. **(C)** Raw input and its reconstruction, computed by summing Poisson-distributed spike trains over the time dimension. **(D)** Weights from input to output neuron populations, initialized initialized from the distribution N(0,1). **(E)** Recurrent weights of the output population, initialized from the distribution $N\left(0,\frac{1}{2}\right)$.

Reservoir computers are typically built from three parts: (1) an encoder that translates input from the environment that is fed to it, (2) a dynamical system based on randomly connected neurons (the *reservoir*), and (3) a readout mechanism. The readout is often trained via gradient descent to perform classification or regression on some target function. BindsNET can be used to build reservoir computers using spiking neurons with little effort, and machine learning functionality from PyTorch can be co-opted to learn a function from states of the high-dimensional reservoir to desired outputs. Code in for defining and simulating a simple reservoir computer is given in Figure 9, and plots to monitor simulation progress are shown in Figure 10. The outputs of the reservoir computer on the CIFAR-10 natural image dataset are used as transformed inputs to a logistic regression model. The logistic regression model is then trained to recognize the categories based on the features produced by the reservoir.

### 4.5. Benchmarking

In order to compare several competing SNN simulators, we devised a simple simulation and benchmarked our software on it against other, similar frameworks. We simulated a network with a population of *n* Poisson input neurons with firing rates (in Hertz) drawn randomly from *U*(0, 100), connected all-to-all with a equally-sized population of leaky integrate-and-fire (LIF) neurons, with connection weights sampled from N(0,1). We varied *n* systematically from 250 to 10,000 in steps of 250, and ran each simulation with every library for 1,000ms with a time resolution *dt* = 1.0. We tested BindsNET (with CPU and GPU computation), BRIAN2, PyNEST (the Python interface to the NEST SLI interface that runs the C++NEST core simulator), ANNarchy (with CPU and GPU computation), and BRIAN2genn (the BRIAN2 front-end to the GeNN simulator). The Nengo and NEURON simulators were considered, but in both cases, we were unable to implement the benchmarked network structure. This speaks to the expressiveness or relative difficulty of using these competing simulation libraries as compared to BindsNET. Several packages, including BRIAN and PyNEST, allow the setting of certain global preferences; e.g., the number of CPU threads, the number of OpenMP processes, etc. We chose these settings for our benchmark study in an attempt to maximize each library's speed, but note that BindsNET requires no setting of such options. Our approach, inheriting the computational model of PyTorch, appears to make the best use of the available hardware, and therefore makes it simple for practicioners to get the best performance from their system with the least effort.

**Figure 11:**
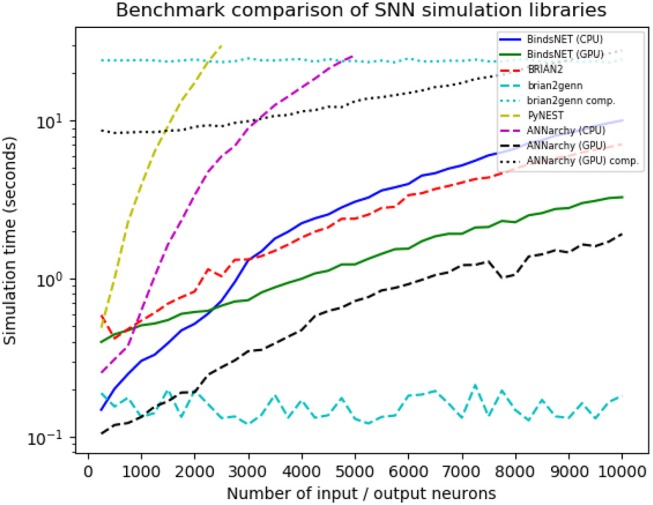
Benchmark comparison results from a number of SNN simulation frameworks. Variability in benchmarked times is likely due to randomness in the simulation and fluctuations in CPU load.

All simulations run on Ubuntu 16.04 LTS with Intel(R) Xeon(R) CPU E5-2687W v3 @ 3.10GHz, 128Gb RAM @ 2133MHz, and two GeForce GTX TITAN X (GM200) GPUs. Python 3.6 is used in all cases except for simulation with ANNarchy, which requires Python 2.7. Clock time was recorded for each simulation run. The results are depicted in Figure 11.

As can be noticed in the Figure 11, PyNEST simulation runs are cut off for *n*> 2.5K, and ANNarchy (on CPUs) for *n*> 5K, due to the fact that, after this point, their simulation time far outstrips those of the other libraries. With small networks (*n* < 2.5K), the CPU-only version of the BindsNET simulation is faster than the BRIAN2 simulation; yet, this relationship reverses as the number of simulated neurons grows. However, in larger networks (*n*> 1.5K), the GPU-only BindsNET simulator is faster than BRIAN2, and is competitive in simulation time in the case of smaller networks. The BRIAN2genn simulator is very fast, with near-constant simulation time of approximately 0.2s; however, it requires a roughly 25s compilation period, no matter the network size, before simulation can begin. Somewhat similarly, simulation with ANNarchy using GPU computation is rather fast, but requires an super-linear increase in compilation time as the size of the network grows.

Therefore, BindsNET constitutes a speed-competitive alternative to several popular existing SNN simulation libraries. Although our benchmark study is far from comprehensive, it demonstrates a particular use case for which BindsNET is perhaps preferable to other methods; i.e., in the case of feedforward networks with all-to-all connectivity. Similar studies can be done to assess its performance relative to the competition in other SNN architectural regimes. We expect that, in different applications, other libraries will perform better in terms of speed or memory usage, and it is up to the experimenter to choose the best software for the simulation task. As stated previously, our approach is best for rapid prototyping and testing of SNNs on CPUs and GPUs alike, which is demonstrated in part by the foregoing benchmark analysis. In particular, a major advantage of using the BindsNET library for GPU computation is that it requires no compilation step intermediate between network definition and simulation, as opposed to the BRIAN2genn and ANNarchy libraries. This is well-suited to machine learning experimentation, which often requires many iterations of model building and hyper-parameter tuning that may be hindered by re-compilation before each attempt.

## 5. Ongoing Developments

BindsNET is still at an early stage of development, and thus there is much room for future work and improvement. Since it is an open source project and because there is considerable interest in the research community in using SNNs for machine learning purposes, we are optimistic that there will be numerous community contributions to the library. Indeed, we believe that public interest in the project, along with the strong support of the libraries on which it depends, will be an important driving factor in its maturation and proliferation of features. We mention some specific implementation goals:

- Additional neuron types, learning rules, datasets, encoding functions, etc. Added features should take priority based on the needs of the users of the library.
- Specialization of machine learning and reinforcement learning algorithms for spiking neural networks. These may take the form of additional learning rules, or more complicated training methods that operate at the network level rather than on individual synapses.
- Tighter integration with PyTorch. Much of PyTorch's neural network functions are useful in the spiking neural network context (e.g., Conv2dConnection), and will benefit from inheriting from them.
- Automatic conversion of deep neural network models implemented in PyTorch or specified in the ONNX format to near-equivalent spiking neural networks (as in Diehl et al., 2015).
- Performance optimization: improving the performance of library primitives will save time on all experiments with spiking neural networks. A high-priority feature is the use of sparse spike vectors and connection weights for efficient linear algebra operations.
- Automatic smoothing of SNNs: approximating spiking neurons as differentiable operations (Hunsberger and Eliasmith, 2015) will enable the use of backpropagation to train networks easily transferable to SNNs. The torch.autograd automatic differentiation library (Paszke et al., 2017) can then be applied to optimize the parameters of spiking networks for ML problems.

## 6. Discussion

We have presented the BindsNET open source package for rapid biologically inspired prototyping of spiking neural networks with a machine learning-oriented approach. BindsNET is developed entirely in Python and is built on top of other mature Python libraries that lend their power to utilize multi-CPU or multi-GPU hardware configurations. Specifically, the ML tools and powerful data structures of PyTorch are a central part of BindsNET's operation. BindsNET may also interface with the gym library to connect spiking neural networks to reinforcement learning environments. In sum, BindsNET represents an additional and attractive alternative for the research community for the purpose of developing faster and more flexible tools for SNN experimentation.

BindsNET comprises a spiking neural network simulation framework that is easy to use, flexible, and efficient. Our library is set apart from other solutions by its ML and RL focus; complex details of the biological neuron are eschewed in favor of high-level functionality. Computationally inclined researchers may be familiar with the underlying PyTorch functions and syntax, and excited by the potential of the third generation of neural networks for ML problems, driving adoption in both ML and computational neuroscience communities. This combination of ML programming tools and neuroscientific ideas may facilitate the further integration of biological neural networks and machine learning. To date, spiking neural networks have not been widely applied in ML and RL problems; having a library aimed at such is a promising step toward exciting new lines of research.

Researchers interested in developing spiking neural networks for use in ML or RL applications will find that BindsNET is a powerful and easy tool to develop their ideas. To that end, the biological complexity of neural components has been kept to a minimum, and high-level, qualitative functionality has been emphasized. However, the experimenter still has access to and control over groups of neurons at the level of membrane potentials and spikes, and connections at the level of synapse strengths, constituting a relatively low level of abstraction. Even with such details included, it is straightforward to build large and flexible network structures and apply them to real data. We believe that the ease with which our framework allows researchers to reason about spiking neural networks as ML models, or as RL agents, will enable advancements in biologically plausible machine learning, or further fusion of ML with neuroscientific concepts.

Although BindsNET is similar in spirit to the Nengo (Bekolay et al., 2014) neural and brain modeling software in that both packages can utilize a deep learning library as a “backend” for computation, Nengo optionally uses Tensorflow in a limited fashion while BindsNET uses PyTorch by default, for all network simulation functionality (with the torch.Tensor object). Additionally, for users that prefer the flexibility and the imperative execution of PyTorch, BindsNET inherits these features and is developed with many of the same design principles in mind. BindsNET has advantages with respect to other simulation libraries using GPU computation, which require costly compilation steps between network building and deployment. BindsNET does not need these expensive intermediate steps as it uses “eager” execution of PyTorch regardless of the actual simulation hardware.

Hardware platforms for spiking neural network computations have advantages over software simulations in terms of performance and power consumption. For example, SpiNNaker (Plana et al., 2011) combines cheap, generic, yet dedicated CPU boards together to create a powerful SNN simulation framework in hardware. Other platforms (e.g., TrueNorth Akopyan et al., 2015, HRL, and Braindrop) involve the design of a new chip. A novel development is Intel's Loihi platform for spike-based computation, outperforming all known conventional solutions (Davies et al., 2018). Other solutions are based on programmable hardware, like FPGAs which transform neural equations to configurations of electronic gates in order to speed up computation. More specialized hardware such as ASIC and DSP can be used to parallelize and therefore accelerate the calculations. In order to conduct experiments in the hardware domain, one must usually learn a specific programming language targeted to the hardware platform, or carefully adapt an existing experiment to the unique hardware environment under the constraints as enforced by chip designers. In either case, this is not an ideal situation for researchers who want rapid prototyping and testing. BindsNET platform introduces a flexibility, which can be exploited in future hardware developments, in particuliar in machine learning problems.

BindsNET is a simple yet attractive option for those looking to quickly build flexible SNN prototypes backed by an easy-to-use yet powerful deep learning library. It encourages the conception of spiking networks as machine learning models or reinforcement learning agents, and is one of the first of its kind to provide a seamless interface with machine learning and reinforcement learning environments. The library is supported by several mature and feature-full open source software projects, and benefits from their growth and continuous improvements. Considered as an extension of the PyTorch library, BindsNET represents a natural progression from second generation neural networks to third generation SNNs.

## Author Contributions

HS and RK initiated the research, produced the conceptual framework, and coordinated the ongoing development efforts. RK and HH conceived and design principles of the BindsNET package. HH and DJS wrote the BindsNET code and the initial version of the manuscript. DJS lead the efforts to create a standardized BindsNET code according to Python specification. HK and DTS helped with improving and testing the BindsNET code. All authors contributed to the revisions and producing the final manuscript.

### Conflict of Interest Statement

The authors declare that the research was conducted in the absence of any commercial or financial relationships that could be construed as a potential conflict of interest.
